# Constructing Interconnected Microporous Structures in Carbon by Homogeneous Activation as a Sustainable Electrode Material for High-Performance Supercapacitors

**DOI:** 10.3390/molecules28196851

**Published:** 2023-09-28

**Authors:** Huijie Li, Rui Ma, Feifei Chen, Danting Wang, Hongmin Zhang, Chunyang Lu

**Affiliations:** 1 School of Surveying and Urban Spatial Information, Henan University of Urban Construction, Pingdingshan 467036, China; lihuijie@huuc.edu.cn; 2 State Key Laboratory of Chemistry and Utilization of Carbon Based Energy Resources, College of Chemistry, Xinjiang University, Urumqi 830017, China; m18699981042@163.com (R.M.); feichen415@163.com (F.C.); wdt0115tt@163.com (D.W.)

**Keywords:** microporous carbon, homogeneous activation, electrode material, supercapacitors

## Abstract

Microporous carbon attracts attention as an electrode material for supercapacitors. However, a large number of deep and distorted mesoporous and macroporous structures are usually created by non-uniform etching, resulting in underutilized internal space. Homogeneous activation has been considered by researchers as a necessary condition for the formation of interconnected microporous structures in carbon materials. Herein, a simple strategy of hydrothermal introduction of defects followed by homogeneous activation for the preparation of microporous carbon was developed for the synthesis of electrode materials for high-performance supercapacitors. The optimized sample with defect-enriched microporous structure and large specific surface area has a specific capacity of 315 F g^−1^ (1 A g^−1^) in KOH solution, and the assembled symmetric supercapacitor achieves a high energy density of 7.3 Wh kg^−1^ at a power density of 250 W kg^−1^. This work is interesting because it not only demonstrates that rational design of electrode materials is important to boost the performance of supercapacitors, but also provides inspiration for the design of efficient supercapacitors in the future.

## 1. Introduction

Supercapacitors are widely researched as energy storage devices in various fields [1,2,3]. Pseudocapacitors and double electric layer supercapacitors (EDLCs) are two different types of supercapacitors that store energy in different ways [4,5,6] and have attracted much attention due to their advantages of high power density, fast charging speed, and long cycle life [7]. Through the double electric layer, EDLCs store energy electrostatically in the form of charges in the electrolyte ions around the electrode–electrolyte contact [8,9]. Pseudocapacitors, on the other hand, utilize reversible and rapid surface redox reactions of the active materials to multiply the charge that has to be stored in the electrodes, but result in reduced power and cycling performance of the device [10,11,12]. Supercapacitors are mainly composed of electrode materials, collectors, a diaphragm between them, and electrolyte. A vital role is played by electrode materials in supercapacitors. Carbon materials, such as carbon nanotubes [13,14,15], porous carbons [16,17], carbon aerogel [18,19], carbon fibers [20,21] and graphene [22,23,24], due to their superior electrical conductivity, pore shape, and extensive supply of precursors, are currently among the most frequently utilized electrode materials. Various raw materials, such as biomass [25,26], coal [27], bitumen [28] or polymers [29,30], are already utilized for the preparation of porous carbon materials by chemical activation or direct carbonization. Wang et al. summarized carbon, inorganic oxides/hydroxides and sulfides, and listed common materials and their electrochemical properties. The author gives a wide range of capacitance distribution, showing that the carbon material has a high specific surface area and excellent chemical stability [31]. ARICÒ et al. reviewed the latest progress in electrolytes and nano-electrode materials for supercapacitors. The advantages and disadvantages of nano-scale in the design of energy storage device materials are emphasized, showing the high capacity and excellent conductivity of carbon materials [32].

Inhomogeneous etching usually produces a large number of deeply layered and tortuous porous structures with a high number of mesopores and macropores, leading to underutilization of internal space. On the contrary, uniform activation provides a pathway for pore modulation, which is accomplished by homogeneous adsorption/coordination of metal ions in the carbon precursor, followed by pyrolysis at elevated temperatures [33,34]. It has been proven that homogeneous activation is a reliable technique for creating interconnected microporous structures with high specific surface area, and it is well-known that the large specific surface area given by the pores makes ion storage easier. Zhang et al. [35]. used simple homogeneous activation of chitosan to design a new microporous carbon with large specific surface area and interconnected porous structure. The sample has a high capacitance retention of 58% at 100 A g^−1^ and a high specific capacitance of 280 F g^−1^/339 F cm^−3^ at 1 A g^−1^. Luo et al. [36] prepared an interconnected microporous capacitive carbon (IMCC) by growing D (+)-glucosamine on bacterial cellulose (BC) nanofibrous scaffolds, followed by carbonization and activation, which facilitated subsequent KOH permeation and homogeneous activation. Utilizing the interconnected microporous structure, the IMCC provided high capacitance of 302 F g^−1^ at 1 A g^−1^ and excellent multiplicative performance of 165 F g^−1^ at 100 A g^−1^ for aqueous supercapacitors. Therefore, it is a key point to inhibit the formation of excessive mesopores and design effective micropores and macropores using homogeneous activation to design porous carbon with high specific surface area.

Due to their renewability and many delicately layered nanostructures or morphologies, biomass-derived porous carbon electrode materials or low-cost natural waste have gained significant attention in the field of energy storage during the past several years. Biomass is highly advantageous as an electrode material, with its own good multidimensional structure and abundant heteroatoms. Although KOH is a widely known efficient activator that generates an abundance of pores during the activation process, its potent etching impact collapses the pore structure, which ultimately causes the sample to degrade. Moreover, the strong corrosion effect of KOH on the equipment renders it unable to be used in the large-scale industrial preparation of biomass-derived porous carbons. HCOOK, as a non-toxic catalyst, has the advantages of being easily soluble in water and ethanol, a readily available material, and inexpensive.

In this study, a novel microporous carbon was homogeneously activated by the green and hypotoxic activator of HCOOK using lavender straw-based hydrochar as the carbon precursor. The hydrothermal pretreatment enriches more active sites for the adsorption of activators, which leads to a homogeneous activation. Hence, the obtained microporous carbon possesses abundant defects, a large specific surface area and an interconnected microporous structure that contributes to fast ionic transport and charge transfer. The experimental results show that the defect-enriched microporous carbon with large specific surface area has a high specific capacitance of 314 F g^−1^ (1 A g^−1^) in KOH solution, and the symmetric supercapacitor can deliver a high energy density of 7.3 Wh kg^−1^ at a power density of 250 W kg^−1^. This work provides a universal and green approach for designing high-performance defect-rich microporous carbon materials using low-cost precursors and offers new insights into high-performance supercapacitors.

## 2. Results and Discussion

**Figure 1:**
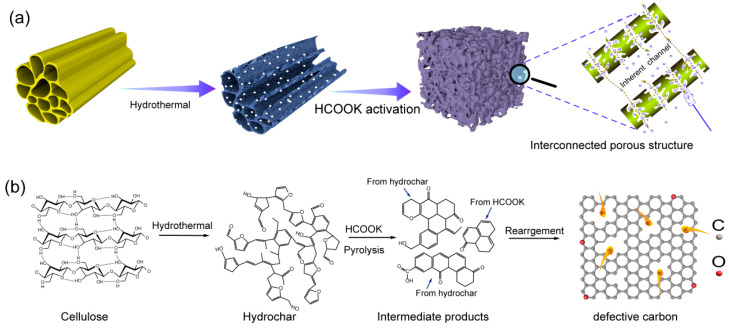
(**a**) Schematic illustrations of the synthesis process for the interconnected microporous carbon. (**b**) Schematic representation of the formation of defective carbon from cellulose.

In a typical process, the lavender straw is firstly subjected to hydrothermal treatment for introducing substantial defects, and then homogeneously activated by HCOOK to obtain microporous dominated interconnected porous carbon. The samples obtained were named XYC-x, where x is a ratio of the mass of HCOOK to the hydrochar. The hydrochar basically inherits the straw morphology after hydrothermalization, but the introduced defects and the exposed oxygen-containing functional groups during the hydrothermal process are preferable to uniformly absorb a great deal of HCOOK, leading to homogeneous activation (Figure 1a). Moreover, the hydrothermal treatment can break the weak chemical bonds in cellulose, causing the formation of small molecules. This ensures a homogeneous mixing of small molecule fragments and HCOOK during the following pyrolysis process and the eventual generation of defect-rich microporous carbon (Figure 1b).

**Figure 2:**
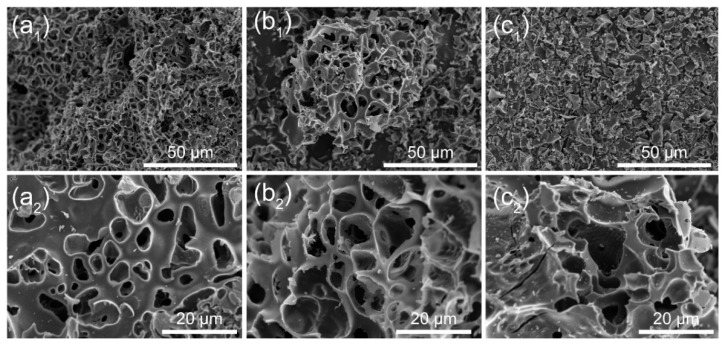
SEM images of (**a_1_**,**a_2_**) XYC-1, (**b_1_**, **b_2_**) XYC-2 and (**c_1_**,**c_2_**) XYC-3.

To understand the underlying mechanisms, we characterized the evolution and morphology of the samples by TEM and SEM. As seen in Figure 2a_1_,a_2_, XYC-1 formed uniform shallow pores on the surface, probably due to incomplete activation of the samples as a result of the low amount of HCOOK. Figure 2b_1_,b_2_ presents regular 3D cross-linked and interconnected porous structures of XYC-2, where pores are absent not only on the surface of the samples, but also show a homogeneous activation of the overall block. From Figure 2c_1_,c_2_, XYC-3 experienced significant pore collapse and block rupture, resulting from the over-activation at high temperatures due to excessive dosing of HCOOK. In addition, TEM images were further tested to observe the morphology and microstructure of XYC-2. Figure S1 shows abundant long-range disordered and short-range ordered regions, revealing the distinct amorphous structure of XYC-2.

For a thorough study of the activation process for the samples in depth, thermogravimetric analysis of hydrothermal carbon (LS), pure HCOOK, and mixtures of the two was carried out under nitrogen atmosphere (Figure S2). The loss of weight of pure LS was comparatively slow before 250 °C, at which time gradual volatilization of organic matter and fracture rearrangement of macromolecules may have occurred. Between 250 °C and 400 °C, the thermogravimetric curve decreased sharply, indicating that the organic materials were partially pyrolyzed. The curve for pure HCOOK only had a remarkable weight loss of about 30% between 400 and 590 °C, demonstrating that HCOOK begins to decompose at 400 °C and breaks down into potassium carbonate at 590 °C. The pyrolysis process of the LS and HCOOK mixture is categorized into three processes: dehydration, carbonization, and activation. The weight loss at low temperatures mainly arises from the decomposition of water and volatile compounds and oxygen-containing groups during the carbonation process. The activation mechanism of HCOOK is shown in Equations (1)–(4). Firstly, HCOOK can decompose to form K_2_CO_3_, H_2_O, and CO_2_. The K_2_CO_3_ reacts with the surrounding carbon atoms (Equation (2)) or directly decomposes to produce potassium oxide and release CO_2_ (Equation (2)). Then, CO_2_ and the in situ produced potassium oxide can react further with the carbon skeleton as shown in Equations (3) and (4) to produce abundant small pores and potassium metal. During the activation process, the metal K vapors can be inserted efficiently into the lattice of the carbon substrate, leading to the expansion of the carbon lattice. During the carbonization and activation process, the continuous release of gases may lead to the forming of more intrinsic carbon flaws in XYC-2 [37,38,39].

2HCOOK → K_2_CO_3_ + H_2_O + CO_2_ (1)

K_2_CO_3_ + C → K_2_O + 2CO (2)

K_2_CO_3_ → K_2_O + CO_2_ (3)

K_2_O + C → 2K + CO (4)

**Figure 3:**
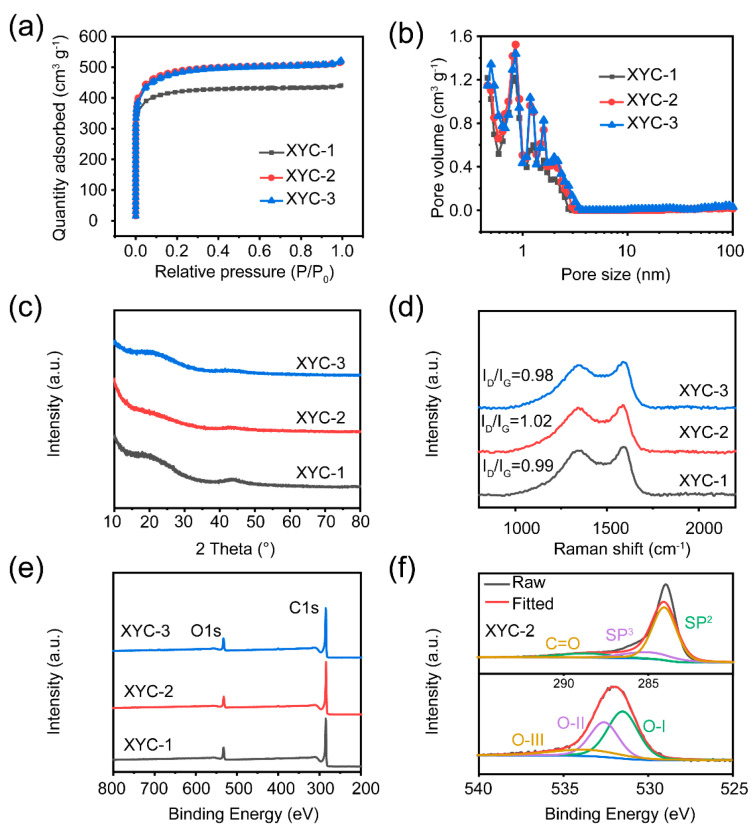
(**a**) N_2_ adsorption-desorption isotherms and (**b**) pore size distribution of the XYCs. (**c**) XRD curves and (**d**) Raman spectra of samples. (**e**) XPS survey spectra of XYCs. (**f**) C1s and O1s spectra of XYC-2.

To further investigate the role of HCOOK activation, the pore size distribution and specific surface area of XYCs were analyzed by N_2_ adsorption and desorption tests. As seen in Figure 3a and Table S1, the XYCs all exhibit type I adsorption isotherms, indicating that the pore structure within the materials is dominated by micropores. The adsorption of the samples rises apparently in the low-pressure region (P/P_0_) and then reaches a distinct plateau, implying that HCOOK activation introduces a significant amount of micropores into the material. With the HCOOK content growing, the results show that the specific surface area of XYC-1 is 1619 m^2^ g^−1^, the specific surface area of XYC-2 is 1834 m^2^ g^−1^, and the specific surface area of XYC-3 is 1802 m^2^ g^−^^1^. This may be attributed to excess HCOOK etching more carbon atoms, resulting in the collapse of existing pores and a reduction of specific surface area. The pore size distribution in Figure 3b further demonstrates that although the pore sizes of the samples increases as HCOOK concentration rises, micropores continue to dominate. In the XRD spectra, the enlargement of the (002) and (100) planes, as well as aberrations and dislocations in the internal lattice of the carbon, were all linked to two broad peaks of the XYCs (Figure 3c). The presence of the most carbon defects in XYC-2 was indicated by the weakest diffraction peaks, which may be a result of the hydrothermal compounds combined with intermediates created by the subsequent pyrolysis of the HCOOK, which introduced many carbon defects and surface oxygen groups [40]. In the Raman spectra illustrated in Figure 3d, all of the samples show distinct D and G peaks located at 1340 cm^−^^1^ and 1580 cm^−^^1^, respectively. The D peak and the G peak indicate the degree of defects and graphitization of the samples, respectively. Generally, the higher I_D_/I_G_ value indicates the more defects there are into the carbon atom crystals. The I_D_/I_G_ value of XYC-2 (1.02) is larger than that of XYC-1 (0.99) and XYC-3 (0.98), indicating that XYC-2 has the most abundant defects. These defects are not only generated by the etching process, but may be attributed to the homogeneous activation of HCOOK introducing some oxygen-containing functional groups [41,42]. Figure 3e shows the XPS spectra of the XYCs samples with two characteristic peaks, namely C1s and O1s at 284.6 eV and 531.6 eV, respectively. The C1s in the XPS spectra can be fitted to three kinds of carbon, which are C=O (288.7 eV), sp3-C (284.9 eV) and sp2-C (284.3 eV) [9]. As can be seen in Table S2, about 88% of the surface atoms of the XYCs are C, and the remaining N and O elements bonded to neighboring atoms enhance the wettability and ion transport of the carbon electrodes. Meanwhile, the rich electrons in the heteroatoms can provide more off-domain electrons for the conjugated carbon skeleton, thus making the electrode highly conductive. The three fitted O1s peaks are carbonyl oxygen (O-I, 531.5 eV), phenol or ether group (O-II, 532.6 eV), and carboxyl or adsorbed water (O-III, 533.7 eV) [8,9]. Table S2 illustrates that C=O carbonyl (O-I) and C-OH hydroxyl or C-O-C ether groups predominate, while O-III peaks denoting oxygen in carboxyl group can also be observed from the surface of carbon materials. In addition to enhancing pseudocapacitance (especially O-I and O-II) in alkaline aqueous electrolyte, the high surface oxygen content also improves the wettability [43,44]. In conclusion, hydrothermal processing can cause more defects (such as internal carbon dislocations, external oxygen doping, etc.) in the carbon structure.

**Figure 4:**
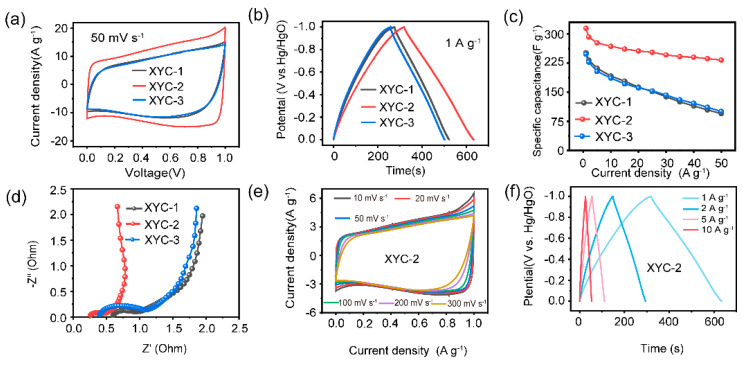
Electrochemical properties of samples measured using the three-electrode system: (**a**) CV curves at 50 mV s^−^^1^. (**b**) GCD curves at 1 A g^−^^1^. (**c**) Rate performance plots of all samples at different current densities. (**d**) Nyquist plots. (**e**) CV curves of XYC-2 at different sweep speeds. (**f**) GCD curves of XYC-2 at different current densities.

Electrochemical tests were first performed on all samples in a three-electrode system to estimate their electrochemical characteristics. The CV curves of the samples are approximately rectangular, which is a typical characteristic of the capacitance of an EDLC (Figure 4a). At the same time, the CV curves also show a hump, which is supposed to be the pseudocapacitance produced by the Faraday reaction. XYC-2 has the largest rectangular region, indicating it possesses the highest specific capacitance. The GCD curves are presented as slightly distorted triangles; among all samples, the XYC-2 exhibits the optimal capacitance performance with the longest discharging time and largest current responsiveness (Figure 4b). The GCD curves can be used to determine the specific capacitances of XYC-1, XYC-2, and XYC-3 at 1 A g^−^^1^, which are 252, 315, and 248 F g^−^^1^, respectively. The findings suggest that the maximum specific capacity value of XYC-2 could be attributed to the reasonable and uniform HCOOK activation producing abundant active sites of the prepared defective carbon, contributing to K^+^ storage. Figure 4c depicts the rate performance of the samples with capacitance retention of 37%, 73.9%, and 40%, respectively, manifesting that XYC-2 has better rate performance than any other sample, with a high current density of 50 A g^−^^1^. As seen in the Nyquist plots from Figure 4d, the straight lines are used to reflect the ion diffusion/migration and capacitance performance of the electrode materials in the low-frequency area. The semicircle represents the charge transfer resistance in the high-frequency zone. The internal resistance of the electrode material, the resistance of the electrolyte, and the contact resistance between the electrode and the current collector form the equivalent series resistance, which is represented by the intercept along the X-axis. XYC-2 has the shortest initial intercept along the X-axis, showing the smallest semicircle diameter and the smallest ohmic resistance (Rs), which indicates its smallest charge-transfer resistance [45,46]. Moreover, the tail of XYC-2 is more vertical in the low-frequency region than that of the other samples, which suggests that it has the optimal EDLC behavior and superior ion transport performance. Moreover, the optimal EDLC behaviors and excellent multiplicity performances of XYC-2 are further confirmed at different sweep speeds (Figure 4e). In particular, the rectangular shape of the CV curve is basically unchanged even when the sweep speed reaches 300 mV s^−^^1^. Figure 4f displays the GCD profiles of XYC-2 with diverse current densities. The GCD curve discharge time is gradually shortened as the current density increases, while maintaining an isosceles triangle shape, further confirming that XYC-2 has high reversibility and stability. The specific capacitance of XYC-2 at 1, 2, 5, and 10 A g^−^^1^ was calculated to be 315, 292, 277, and 268 F g^−^^1^, respectively, much higher than XYC-1 and XYC-3 measured under the same current density.

**Figure 5:**
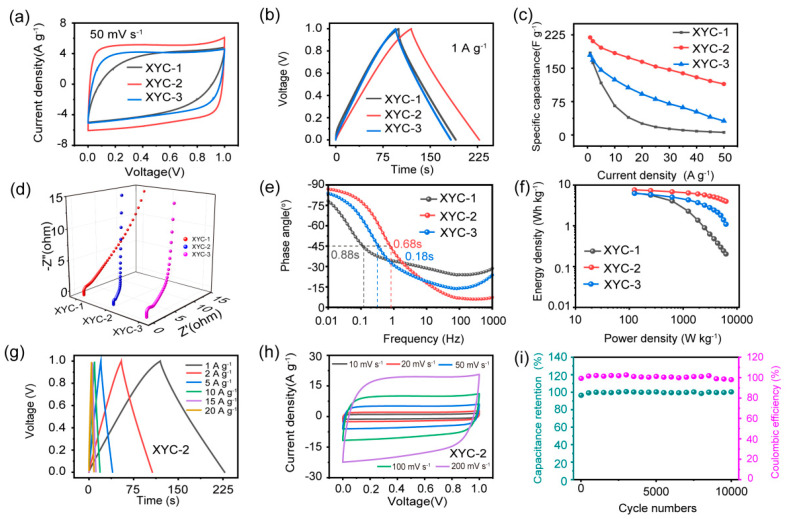
Electrochemical properties of samples measured using a two-electrode system: (**a**) CV curve at 50 mV s^−^^1^. (**b**) GCD curve at 1 A g^−^^1^. (**c**) Plot of rate performance at different current densities. (**d**) Nyquist plots. (**e**) Bode plots. (**f**) Ragone plots of samples. CV curves of XYC-2 at different scan rates. (**g**) GCD curves of XYC-2 at different current densities. (**h**) CV curves. (**i**) Cycling performance of XYC-2.

To further verify their performance in practical applications, symmetric supercapacitors were assembled with traditional electrolyte based on XYC-1, XYC-2, and XYC-3. Figure 5a,b shows that, compared with XYC-1 and XYC-3, XYC-2 has the longest discharge time at 1 A g^−1^and the largest current response at 50 mV s^−1^. A a current density of 1 A g^−1^, it possesses specific capacitance of 220 F g^−1^, indicating the optimal capacitive characteristic. As shown in Figure 5c, the capacitance retention of XYC-2 at 50 A g^−1^ is 52%, demonstrating its superior rate performance over XYC-1 and XYC-3. At a high scan rate of 200 mV s^−1^, the rectangular shapes of the CV curves are as illustrated in Figure 5d. The near-triangular shapes of the constant-current charge/discharge curves at different currents further demonstrate that XYC-2 has a good EDLC storage performance with a low ion-transport resistance. The Bode plots of the XYCs are showed in Figure 5e. The phase angle of XYC-2 is 87°, nearing the ideal capacitance (90°), which may be attributed to the large number of carbon defects introduced by the hydrothermal process and the following homogeneous activation. The frequency at a phase angle of 45° was used to derive the relaxation time constant (f_0_), which was calculated as τ_0_ = 1/f_0_. This value for XYC-2 is 0.18 s, which is the quickest relaxation time when compared to the other samples. This result also shows that electrolyte ions are rapidly adsorbing and diffusing on the internal pores of the XYC-2 electrode. The energy density versus power density of symmetric supercapacitors based on XYCs was calculated from the GCD curves and plotted on a Ragone diagram (Figure 5f). XYC-2 achieves an energy density of 7.3 Wh kg^−1^ at a low power density of 250 W kg^−1^, outperforming XYC-1 (5.8 Wh kg^−1^) and XYC-3 (5.6 W h kg^−1^). The GCD curve remains triangular at high current density (20 A g^−1^), and the CV curve remains rectangular at high sweep rate (200 mV s^−1^), further exhibiting the excellent reversibility and rate performance of XYC-2. Figure 5g shows the GCD curves and CV curves of the symmetric supercapacitor, which indicates the excellent reversibility and stability of the sample. Additionally, a specific capacitance retention of 99% and a Coulombic efficiency of approximately 100% were attained after 10,000 cycles at 5 A g^−1^ (Figure 5i), confirming the exceptional electrochemical stability and excellent reversibility of XYC-2.

## 3. Materials and Methods

Lavender straw from Yili in Xinjiang province was used in this experiment. The straw was rinsed with distilled water to remove surface ash and heated in an oven at 80 °C. Then, the straw was crushed using an ultra-high-speed pulverizer. The lavender straw powder was pretreated with water as a solvent at 180 °C for 12 h (denoted as LS), then homogeneously mixed with HCOOK in different ratios, transformed in a corundum crucible, and heated to 800 °C for 2 h in a tube furnace with an N_2_ atmosphere. At the end of the reaction, the crucible was firstly acid-washed with HCl several times. Then, the acid-washed samples were washed to neutral with deionized water, dried and ground to prepare porous carbon. Finally, the dried samples were named XYC-1, XYC-2, and XYC-3, where 1, 2, and 3 represent the mass ratios of HCOOK to the hydrochar, respectively.

The active ingredient, acetylene black and polytetrafluorethylene (PTFE) in an 8:1:1 mass ratio were combined uniformly in ethanol to prepare working electrodes in an 8:1:1 mass ratio. The dried and cut flakes were then pressed onto nickel foam to prepare the working electrodes. Each working electrode had a mass loading of 3 mg cm^−^^2^ active material. A three-electrode system was applied to estimate the electrochemical performance, with a Pt electrode serving as the counter electrode and 6 M KOH solution and Hg/HgO serving as the electrolyte and reference electrode, respectively. Using a diaphragm to separate the two identical porous carbon working electrodes and 6 M KOH solution as the electrolyte, the symmetric supercapacitor was assembled in a CR2032 coin cell.

### 3.1. Electrochemical Measurements

Electrochemical impedance spectroscopy (EIS), galvanostatic charge–discharge (GCD), and cyclic voltammetry (CV) measurements were conducted in an electrochemical workstation (CHI 660E, Chenhua, Shanghai, China). In order to compare with the reported results, we calculated the specific capacitance of the supercapacitor using GCD and CV curves, respectively. The specific capacitance (C_s_, F g^−^^1^) of the two-electrode system and the three-electrode system based on the active substance was calculated according to the following equations, respectively:

C_s_ = IΔt/mV (5)

C_s_ = 2IΔt/mV (6)

where I (A) is discharge current, Δt (s) is discharge time, m (g) is the mass of active materials and V (V) is working voltage.

The energy density E (Wh kg^−^^1^) and power density P (W kg^−1^) were calculated by the following equations, respectively:

E = C_s_ × ΔV^2^/28.8 (7)

P = 3600 E/Δt (8)

where ΔV (V) is the working voltage excluding IR drop.

### 3.2. Structural Characterization

Cu K radiation was used to obtain X-ray diffraction (XRD, Bruker D8 Advance, Germany). Surface elemental compositions, bonding states, and functional groups were determined by X-ray photoelectron spectroscopy (XPS, Thermo, ESCALAB 250xi, Waltham, MA, USA). Using scanning electron microscopy (SEM, Hitachi, S-4800, Tokyo, Japan) and transmission electron microscopy (TEM, JEOL, 2100F, Tokyo, Japan), the morphology and microstructure of the samples were examined. Pore size distribution and specific surface area were obtained with an N_2_ adsorption/desorption analyzer (Micromeritics, ASAP 2020, Norcross, GA, USA). Raman spectroscopy was performed with a confocal microscope Raman spectrometer (Renishaw, in Via Reflex Britain).

## 4. Conclusions

In summary, a simple strategy of hydrothermal introduction of defects followed by homogeneous activation for the preparation of microporous carbon was developed in this work for the synthesis of electrode materials for high-performance supercapacitors. The ion migration kinetics were greatly improved by encouraging the creation of short, interconnecting microporous structures, which significantly enhanced the ion migration kinetics. In a three-electrode system, the optimized XYC-2 reflects superior multiplicity performance and a high specific capacitance of 315 F g^−^^1^. Assembled into a symmetric supercapacitor, the capacity retention was 99% after 10,000 cycles. The results are expected to provide guidance for the fabrication of innovative electrode materials in supercapacitors.

## Data Availability

Data are contained within the article.

## Supplementary Materials

The following supporting information can be downloaded at: https://www.mdpi.com/article/10.3390/molecules28196851/s1.

## Sample Availability

Samples of the compounds are available from the authors.
