# Supplementary material for: Constructing Interconnected Microporous Structures in Carbon by Homogeneous Activation as a Sustainable Electrode Material for High-Performance Supercapacitors
Source: Molecules. 2023 Sep 28;28(19):6851. doi: 10.3390/molecules28196851 (PMC10574793; doi:10.3390/molecules28196851)
Supplement: Supplementary file 1 [file molecules-28-06851-s001.zip › molecules-2620767-supplementary.pdf]

Supporting information

Constructing interconnected microporous structure in carbon by homogeneous activation as a sustainable electrode material for high-performance supercapacitors

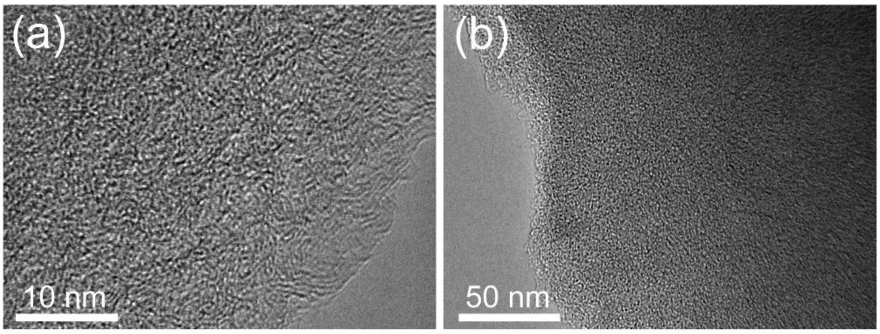

Figure S1. TEM images of XYC-2

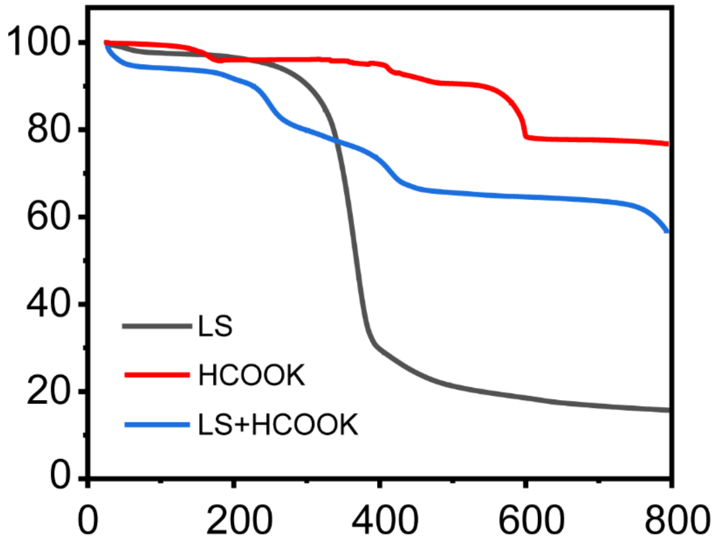

Figure S2. (a) TG curves of different samples

Table S1. Pore structure parameters of as-obtained samples.

sample	SSA (m^2 g^{-1})			Pore volume (cm^3 g^{-1})		
	S_{BET}	S_{micro}	S_{micro}/S_{BET}	V_{total}	V_{Micro}	D_{aver} (nm)
XYC-1	1619	1366	0.84	0.66	0.53	1.66
XYC-2	1834	1480	0.81	0.86	0.59	1.71
XYC-3	1802	1383	0.77	0.68	0.56	1.75

S_{BET}: The specific surface area calculated by BET method;

S_{micro}: t-Plot Micropore Area;

V_{total}: Single point adsorption total pore volume at P/Po = 0.995;

V_{micro}: t-Plot Micropore Area;

D_{aver}: Average pore width calculated by $4V_{total}/S_{BET}$.

Table S2. Surface chemical compositions of the prepared samples

Sample	C (at%)	O (at%)	O-I (at%)	O-II (at%)	O-III (at%)
XYC-1	87.98	10.42	3.25	4.51	2.66
XYC-2	88.97	9.28	5.48	2.31	1.49
XYC-3	88.26	9.91	2.91	4.55	2.45
